# Effects of lycopene on abdominal fat deposition, serum lipids levels and hepatic lipid metabolism-related enzymes in broiler chickens

**DOI:** 10.5713/ajas.20.0432

**Published:** 2020-10-13

**Authors:** Xiaoli Wan, Zhengfeng Yang, Haoran Ji, Ning Li, Zhi Yang, Lei Xu, Haiming Yang, Zhiyue Wang

**Affiliations:** 1 College of Animal Science and Technology, Yangzhou University, Yangzhou, Jiangsu 225009, China; 2 Joint International Research Laboratory of Agriculture and Agri-Product Safety of Ministry of Education of China, Yangzhou University, Yangzhou, Jiangsu 225009, China

**Keywords:** Lycopene, Abdominal Fat, Serum Lipid, Lipid Metabolism, Broiler Chicken

## Abstract

**Objective:**

The present study was conducted to investigate the effects of lycopene on growth performance, abdominal fat deposition, serum lipids levels, activities of hepatic lipid metabolism related enzymes and genes expression in broiler chickens.

**Methods:**

A total of 256 healthy one-day-old male Arbor Acres broiler chicks were randomly divided into four groups with eight replicates of eight birds each. Birds were fed basal diet supplemented with 0 (control), 100, 200, and 400 mg/kg lycopene, respectively.

**Results:**

Dietary 100 mg/kg lycopene increased the body weight at 21 day of age compared to the control group (p<0.05). Compared to the basal diet, broilers fed diet with 100 mg/kg lycopene had decreased abdominal fat weight, and broilers fed diet with 100 and 200 mg/kg lycopene had decreased abdominal fat percentage (p<0.05). Compared to control, diets with 100, 200, and 400 mg/kg lycopene reduced the levels of total triglyceride and total cholesterol in serum, and diets with 100 and 200 mg/kg lycopene reduced the level of serum low density lipoprotein cholesterol (p<0.05). The activity of fatty acid synthase (FAS) in 400 mg/kg lycopene treated broilers and the activity of acetyl-CoA carboxylase (ACC) in 100, 200, and 400 mg/kg lycopene treated broilers were lower than those fed basal diet (p<0.05). Lycopene increased the mRNA abundance of adenosine monophosphate activated protein kinase α (AMPK-α), whereas decreased the mRNA abundance of sterol regulatory element-binding protein 1, FAS, and ACC compared to the control group (p<0.05).

**Conclusion:**

Dietary lycopene supplementation can alleviate abdominal fat deposition and decrease serum lipids levels, possibly through activating the AMPK signaling pathway, thereby regulating lipid metabolism such as lipogenesis. Therefore, lycopene or lycopene-rich plant materials might be added to poultry feed to regulate lipid metabolism.

## INTRODUCTION

The broiler industry has thrived, and broiler chicks have become one of the common meat species during the past few decades. However, excessive deposition of abdominal and subcutaneous fat resulting from the intensive production, not only leads to high occurrence of ascites syndrome and sudden death syndrome, but also has a negative impact on consumers’ healthy diet [1]. Studies have shown that dietary supplementation of specific natural or synthetic compounds had lipid lowering effect [2–4]. Consumers increasingly prefer natural to synthetic compounds in consideration of food safety and health. Thus, plant derived compounds or phytogenic products used as feed additives have received quite a bit of attention [5].

With the increase of natural source additives used in animal diets, carotenoids such as lycopene are gradually drawing the attention of the feed industry. Lycopene, which is a natural isoprene compound belonging to carotenoids, is present in vegetables, fruits, and flowers with red, yellow, or orange colors, especially in tomatoes, carrots, watermelons, pawpaws [6,7]. Furthermore, lycopene is a natural and efficient pigment used in food processing, and has the bioactivity of antioxidant activity, immunoregulation, anticancer, blood lipids regulation and cardiovascular diseases prevention effects [7]. It has been identified as a class A nutrient by the United Nations Food and Agriculture Organization (FAO) and World Health Organization (WHO) [7]. Although lycopene is known for its antioxidant activity, the blood lipids regulation capacity is not neglectable. Studies in rats, mice or humans have shown that lycopene and lycopene enriched tomato products could reduce the risk of obesity and cardiovascular disease by regulating lipid metabolism [8–10]. Lycopene or lycopene-enriched plant materials in poultry diet also showed positive regulation effect on serum lipids levels [11–14].

In this study, we investigate the effects of dietary lycopene supplementation on abdominal fat deposition, serum lipids levels, activities of hepatic lipid metabolism enzymes, as well as mRNA expression levels of related enzymes and transcription factors in liver, to elucidate whether lycopene could influence lipid metabolism in broiler chickens. The results of this study will provide information for the application of lycopene-rich plant materials in animal feed.

## MATERIALS AND METHODS

### Animal and management

All animal experiments were conducted in accordance with the Institutional Animal Care and Use Committee of Yangzhou University, Yangzhou, China (No. SYXK(Su)2016-0020).

A total of 256 healthy one-day-old male Arbor Acres broilers chicks were obtained from a commercial hatchery (Nantong, Jiangsu Province, P. R. China). All birds were randomly divided into four groups with eight replicates per group, each replicate comprised eight birds, and kept in an environmentally controlled room. The room temperature was 32°C to 34°C for the first three days, then gradually decreased to 22°C±1°C by 2°C to 3°C per week and maintained until the end of the experiment. Broilers in the control group were offered the basal diet, whereas broilers in the other three groups were fed the basal diet supplemented with 100, 200, and 400 mg/kg lycopene, respectively. Lycopene (80% of purity) was purchased from Shanghai yuanye Bio-Technology Co., Ltd (Shanghai, China). The basal corn-soybean meal diet was formulated based on the recommendation of NRC (1994) to meet the nutrient requirement of broilers (Table 1). All birds in the experimental period (1 to 42 day of age) enjoyed free access to feed and water with a lighting cycle of 23 h light and 1 h dark. The management procedures were conducted based on the commercial chicken farm feeding management procedures. Body weight (BW) and feed intake (FI) were recorded at 21 and 42 day of age by replicate and gain to feed ratio (G/F) was calculated by the ratio between body weight gain (BWG) and FI.

### Sample collection

At the end of the experiment, one bird per replicate with an averaged BW of the replicate was selected after 12 h feed deprivation. Blood sample was obtained from the wing vein, then the serum was collected after centrifuged at 3,000×*g* for 10 min at 4°C. The serum samples were stored at −20°C for total triglyceride (TG), total cholesterol (TC), low density lipoprotein cholesterol (LDLC) and high density lipoprotein cholesterol (HDLC) analysis. After bleeding, the broilers were euthanized by cervical dislocation. Immediately after death, the abdominal fat of each broiler was harvested and weighed. The liver samples were excised, washed with ice-cold sterilized saline, then snap-frozen in liquid nitrogen, and stored at −70°C before use.

### Determination of abdominal fat deposition

The abdominal adipose deposition was expressed as abdominal fat percentage (AFP), which was calculated by the percentage of abdominal fat weight (AFW) to final BW.

### Measurement of serum TG, TC, LDLC, and HDLC levels

The serum TC, TG, LDLC, and HDLC levels were determined using an automatic biochemical analysis system (UniCel Synchron DxC 800 synchron, Beckman Coulter, Inc., Fullerton, CA, USA). The results were expressed as millimole per litre (mmol/L).

### Hepatic enzymatic activity

The commercial enzyme-linked immunosorbent assay kits of Shanghai Yu Bo Biotech Co., Ltd (Shanghai, China) were used to measure the hepatic activities of fatty acid synthase (FAS), acetyl-CoA carboxylase (ACC), lipoprotein lipase (LPL), and hepatic lipase (HL), and operated according to the instructions of the corresponding kits. The bicinchoninic acid assay was used to measure the protein concentration of liver [15]. The results were calculated based on the protein concentration of liver and expressed as units per gram of protein (U/g protein).

### mRNA quantification

Total RNA isolation of liver sample was performed following the protocol of Uni-10 column Trizol total RNA extraction kit (Sangon Biotech Co. Ltd., Shanghai, China). The quality of the RNA was performed by agarose gel electrophoresis detection, and the quantity of the RNA was measured with a microspectrophotometer (NaroDrop 2000c, Thermo Scientific, Waltham, MA, USA).

Immediately following the total RNA isolation, reverse transcription was performed using the PrimeScript RT Master Mix kit (TaKaRa Biotechnology Co. Ltd., Dalian, China) to synthesis cDNA. Real-time polymerase chain reaction (PCR) was performed with the 7500 Real-Time PCR System (Applied Biosystems, Foster City, CA, USA) with a TB Green P*remix Ex Taq* II kit (TaKaRa, China) following the instruction of the kit. The primer sequences for broiler adenosine monophosphate activated protein kinase α (*AMPKα*), sterol regulatory element-binding protein 1 (*SREBP-1*), *FAS*, *ACC*, and β-actin (as a reference gene) are listed in Table 2. The primers were synthesized by Sangon Biotech Co. Ltd (China). The relative expression level of each target gene mRNA was normalized to the mRNA level of β-actin, and the results were expressed as the fold difference between the treated groups and the control group using the 2^−ΔΔCt^ method [16].

### Statistical analysis

Data are presented as mean±standard error of the mean, and statistical analyzed by SPSS (version 22 for windows, SPSS Inc, Chicago, IL, USA). Data were subjected to one-way analysis of variance using the general liner model procedure of SPSS, and Tukey test was used to assess the differences among the treatment means. Differences were considered significant when p<0.05.

## RESULTS

### Growth performance

Effects of various dietary lycopene supplementation on growth performance of broiler chickens are shown in Table 3. Compared to the control group, diet supplemented with 100 mg/kg lycopene increased the BW at 21 day of age (p<0.05). Dietary lycopene addition had no significant impact on FI, G/F, BWG from 22 to 42 days of age and BW at 42 day of age (p> 0.05).

### Abdominal fat deposition

Table 4 shows the effects of dietary lycopene inclusion on the AFW and AFP of broiler chickens. The AFW of broilers treated by 100 mg/kg lycopene was decreased in comparison to the basal diet (p<0.05). The AFP of broilers in 100 and 200 mg/kg lycopene supplementation groups were lower than that of the control group (p<0.05).

### Serum lipid level

The serum lipid parameters are presented in Table 5. Broilers fed diets supplemented with 100, 200, and 400 mg/kg lycopene reduced the levels of TG and TC in serum compared to control (p<0.05). Broilers fed diets supplemented with 100 and 200 mg/kg lycopene reduced the level of serum LDLC compared to control (p<0.05).

### Hepatic enzymatic activity

As shown in Table 6, the hepatic activity of FAS in the 400 mg/kg lycopene group was lower than control group (p< 0.05). The activity of ACC in the 100, 200, and 400 mg/kg lycopene treated broilers were lower than broilers fed basal diet (p<0.05). However, the activities of LPL and HL were similar among all experimental groups (p>0.05).

### Relative mRNA expression level of gene

**Figure 1:**
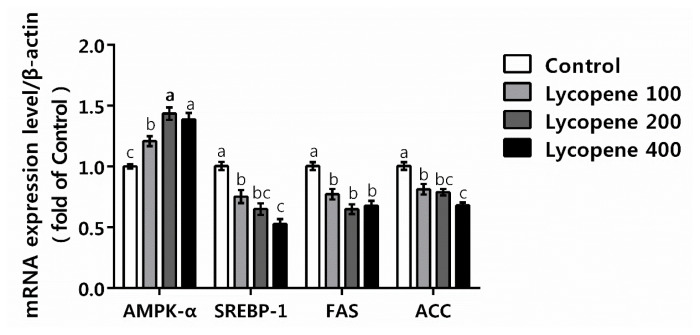
Effects of lycopene on hepatic mRNA expression level of genes in broiler chickens. Values are presented means±standard error of the means, n = 8. Bars with different letters differ significantly (p<0.05). Control, broiler chickens fed basal diet; Lycopene 100, broiler chickens fed basal diet supplemented with 100 mg/kg lycopene; Lycopene 200, broiler chickens fed basal diet supplemented with 200 mg/kg lycopene; Lycopene 400, broiler chickens fed basal diet supplemented with 400 mg/kg lycopene; AMPKα, adenosine monophosphate activated protein kinase α; SREBP-1, sterol regulatory element-binding protein 1; FAS, fatty acid synthase; ACC, acetyl-CoA carboxylase.

To investigate the potential mechanism of lycopene in lowering lipid, the gene expression of some key enzymes and factors in the hepatic lipid metabolism were determined. The mRNA expression levels of some genes that significantly changed can be seen from Figure 1. The mRNA expression level of AMPK-α in lycopene supplemented groups were increased compared to the control group (p<0.05). Whereas, the mRNA expression levels of SREBP-1, FAS and ACC in lycopene supplemented groups were decreased compared to the control group (p<0.05).

## DISCUSSION

A previous study suggested that broiler chickens’ diet with added 100 mg/kg lycopene from 14 to 35 days of age enhanced the final live weight [11]. According to the study of Lira et al [17], tomato waste might be used as an ingredient of broiler chickens’ diet (up to 20%) from 29 to 42 days of age with no negative effect on growth performance. Diet supplemented with 5% dried tomato pomace (lycopene enriched) increased body mass and decreased FI to weight gain ratio of broilers at 28 day of age [12]. Results in the present study showed that lycopene was beneficial to the early growth of broiler chickens. Although some discrepancies exist among these abovementioned studies due to species, ages, doses, or duration, it is convincible that there is no negative effect of lycopene on growth of broilers. Therefore, lycopene or lycopene enriched materials can be used as additives or ingredients in poultry feed.

Abdominal adipose mass is an important index used to measure lipid deposition in broilers. Serum TG, TC, LDLC, and HDLC levels are essential biochemical parameters reflecting the lipid metabolism status. TG, TC, and LDLC have been established as risk factors and elevated HDLC level possesses protective properties. Our present study revealed that dietary lycopene addition decreased abdominal fat mass, and reduced serum TG, TC, and LDLC levels. Similarly, lycopene enriched tomato-wine raised HDLC level and produced anti-obesity impact through inhibition of lipid biosynthesis in high-fat diet (HFD) fed rats [9]. Rats ingesting virgin olive oil and argan oil with lycopene had reduced serum TG, TC, LDLC concentrations and elevated serum HDLC concentration, and decreased hepatosomatic index, these indicated that lycopene could fight against hyperlipidemic and hypercholesterolemic-derived disorders [18]. Lycopene and tomato powder (both provided 10 mg lycopene/kg food/d) supplementation in mice diet reduced serum TG concentration, prevented HFD-induced hepatic steatosis and hypertrophy of adipocytes [19]. Dietary 200 mg/kg lycopene supplementation in Feedlot Bamei Lamb reduced the levels of plasma TG, TC, and LDLC, as well as atherogenic index, and the levels of TG and LDLC were decreased with the feeding time extension [20]. Similar results were observed in poultry. Broilers fed diet supplemented with 5% dried tomato pomace (708 mg lycopene/kg diet) from 1 to 28 days had lower TG and higher HDLC concentrations in serum than the basal diet [12]. Dietary supplementation of 50, 100, and 200 mg/kg lycopene increased the HDLC concentration, whereas decreased TC, TG, and LDLC concentrations in Japanese quails [13]. A previous study of Sun et al [14] revealed that 40 mg/kg lycopene supplementation in chick diet increased serum HDLC level. The results observed in the present study agree with the abovementioned literatures [12–14,19,20], suggesting that lycopene or lycopene enriched tomato product could regulate serum lipids levels in poultry. Diet with tomato juice could inhibit the activity of cholesterol synthesis rate-limiting enzyme, 3-hydroxy-3-methylglutaryl coenzyme A reductase (HMGCR) [21]. The possible mechanism in the effect of lowering cholesterol of lycopene and tomato derivatives possibly via inhibiting the activity and expression of HMGCR, modulating the low density lipoprotein receptor (LDL-R) and inhibiting the activity of acyl-coenzyme A:cholesterol acyltransferase (ACAT) [22]. Therefore, lycopene might have a role in lipid metabolism.

In avian species, the liver accounts for about 95% of de novo fatty acid synthesis, making it the main organ of lipogenesis, and it is generally considered that most of the lipid that accumulate in adipose tissue is synthesized in the liver or provided by diet fat [23]. The liver is often used as target organ in the scientific research of exogenous materials treat diabetes and obesity. Therefore, regulation of the hepatic lipogenesis or steatolysis enzymes in relevant metabolic pathways play essential roles in lipid metabolism. The AMPK is a central regulator of energy metabolism and plays a crucial role in lipid metabolism regulation [24]. AMPK consists of three subunits (α, β, γ), α is the catalytic subunit, β and γ are the regulatory subunits, and α subunit is essential for AMPK activation [25]. In liver, the lipid metabolism regulation effect of AMPK acts through the regulation of SREBP-1 [26]. SREBP-1 is a key transcription factor in cholesterol and fatty acid biosynthesis, which is preferentially involved in the regulation of genes related to fatty acid synthesis, can directly stimulate the transcription of genes encoding FAS [27], and modulate the expression of enzymes involved in the synthesis of fatty acid, cholesterol, triacylglycerol, and phospholipid [28]. The activation of AMPK decreased the activity of FAS through downregulating the mRNA expression of SREBP-1, thereby inhibiting lipogenesis [29]. In addition, the activation of AMPK phosphorylates/inhibits ACC results in reduced malonyl-CoA, then inhibits the synthesis of fatty acid [24]. Plant-derived phytochemicals are indirect activators of AMPK [24]. Zeaxanthin, a kind of carotenoid, could play anti-obesity role by activating AMPK, thereby inhibiting lipogenesis [30]. Fenni et al [19] have recently shown that lycopene and tomato powder (both provided 10 mg lycopene/kg food/d) supplementation in HFD fed mice decreased the mRNA expression of hepatic ACC, FAS, and SREBP-1c. Similarly, in the present study, lycopene addition in broilers diet decreased the activities of FAS and ACC, upregulated the mRNA expression level of AMPKα, down regulated the mRNA expression levels of SREBP-1, FAS, and ACC in liver. Thus, it is convincible that broilers fed diet supplemented with lycopene might activate the AMPK signaling pathway, thereby inhibiting lipid synthesis in liver. The reduced abdominal fat deposition and regulated serum lipid level might partly be attributable to the activated AMPK signaling pathway.

## CONCLUSION

Compared to basal diet, dietary lycopene supplementation decreased abdominal fat deposition, reduced serum TG, TC, and LDLC levels, and reduced hepatic FAS and ACC activities of broilers. Furthermore, lycopene might activate the AMPK signaling pathway, thereby modulating lipid metabolism such as lipogenesis. The beneficial effects of lycopene appeared to be mainly mediated through inhibition of hepatic lipid biosynthesis, and the inclusion level of 100 mg/kg lycopene in broiler diet was recommended in this study. These findings can benefit our understanding on the supplementation of lycopene as a potential precaution to protect against hyperlipidemia, excessive abdominal fat deposition and associated diseases in poultry, as well as provide information for the application of lycopene-rich plant materials in animal feed.

## Figures and Tables

**Table 1:** Composition and nutrient level of basal diet (as fed basis, %)

Items	1 to 21 d	22 to 42 d
Ingredients		
Corn	57.10	61.00
Soybean meal	31.00	28.00
Corn gluten meal	4.00	1.60
Soybean oil	3.00	1.30
Dicalcium phosphate	2.00	2.40
Limestone	1.20	4.00
L-lysine	0.20	0.25
DL-methionine	0.20	0.15
Premix1)	1.00	1.00
Sodium chloride	0.30	0.30
Total	100.00	100.00
Calculated nutrient levels		
Apparent metabolizable energy (MJ/kg)	12.61	12.96
Crude protein	21.36	19.44
Calcium	1.00	0.93
Available phosphorus	0.46	0.39
Lysine	1.09	1.05
Methionine	0.56	0.47
Arginine	1.27	1.16
Methionine+cystine	0.91	0.80

1) The premix provided per kilogram of diet: vitamin A (retinyl acetate), 12,000 IU; vitamin D_3_ (cholecalciferol), 2,500 IU; vitamin E (DL-α-tocopheryl acetate), 20 IU; menadione, 1.3 mg; thiamin, 2.2 mg; riboflavin, 8.0 mg; nicotinamide, 40 mg; choline chloride, 400 mg; calcium pantothenate, 10 mg; pyridoxine HCl, 4 mg; biotin, 0.04 mg; folic acid, 1 mg; vitamin B_12_ (cobalamin), 0.013 mg; Fe (from ferrous sulfate), 80 mg; Cu (from copper sulphate), 8.0 mg; Mn (from manganese sulphate), 110 mg; Zn (from zinc sulfate), 60 mg; I (from calcium iodate), 1.1 mg; Se (from sodium selenite), 0.3 mg.

**Table 2:** Primer sequences used for real-time polymerase chain reaction

Gene ID	Accession ID	Gene name	Primer sequence 5′-3′	Product length (bp)
396526	NM_205518	*β-actin*	Forward: TGATATTGCTGCGCTCGTTG Reverse: ATACCTCTTTTGCTCTGGGCTT	183
427185	NM_001039603	*AMPK-α*	Forward: GATATTTGGAGCAGTGGGGTTA Reverse: GGAAACAAGTATTTGGGAAGGT	248
373915	NM_204126	*SREBP-1*	Forward: ACCGCTGCTTCCACTTCC Reverse: TGAGCCAACGGGTCCACT	208
396061	NM_205155	*FAS*	Forward: GGTGGAAGCCAGGGATTTA Reverse: ACGGAGGGCAGTTGGATTA	188
396504	NM_205505	*ACC*	Forward: GCTGGGTTGAGCGACTAATG Reverse: AAACTGGCAAAGGACTGACG	170

*AMPKα*, adenosine monophosphate activated protein kinase α; *SREBP-1*, sterol regulatory element-binding protein 1; *FAS*, fatty acid synthase; *ACC*, acetyl-CoA carboxylase.

**Table 3:** Effects of lycopene on growth performance of broiler chickens

Items	Control	Lycopene (mg/kg)			p-value

		100	200	400	
1–21 day of age					
21 day BW (g)	679.77±7.43^b^	733.59±16.28^a^	723.79±9.77^ab^	706.79±12.07^ab^	0.018
FI (g)	1,010.48±14.05	1,055.75±19.09	1,059.62±23.43	1,038.67±21.64	0.305
G/F	0.633±0.004	0.656±0.008	0.646±0.006	0.642±0.004	0.080
22–42 day of age					
BWG (g)	1,475.76±7.14	1,507.65±21.80	1,545.02±25.22	1,522.47±26.64	0.170
FI (g)	2,847.71±24.77	2,873.41±33.89	2,963.34±28.82	2,920.07±37.99	0.072
G/F	0.518±0.004	0.525±0.004	0.521±0.006	0.521±0.004	0.793
1–42 day of age					
42 day BW (g)	2,155.53±12.79	2,241.24±34.45	2,268.81±33.39	2,229.25±35.17	0.078
FI (g)	3,858.19±34.33	3,929.16±48.23	4,022.96±44.24	3,958.73±47.80	0.088
G/F	0.548±0.003	0.560±0.003	0.554±0.003	0.553±0.003	0.121

Values are presented means±standard error of the means, n = 8.

BW, body weight; FI, feed intake; G/F, gain to feed ratio; BWG, body weight gain.

a,b Means within a row with different superscripts differ significantly (p<0.05).

**Table 4:** Effects of lycopene on abdominal fat deposition of broiler chickens

Items	Control	Lycopene (mg/kg)			p-value

		100	200	400	
AFW (g)	28.69±1.62^a^	22.50±0.97^b^	24.89±1.34^ab^	25.61±1.23^ab^	0.021
AFP (%)	1.37±0.05^a^	1.06±0.04^b^	1.16±0.06^b^	1.19±0.06^ab^	0.004

Values are presented means±standard error of the means, n = 8.

AFW, Abdominal fat weight; AFP, Abdominal fat percentage.

a,b Means within a row with different superscripts differ significantly (p<0.05).

**Table 5:** Effects of lycopene on serum lipid level of broiler chickens

Items	Control	Lycopene (mg/kg)			p-value

		100	200	400	
TG (mmol/L)	1.12±0.06^a^	0.70±0.04^b^	0.71±0.03^b^	0.61±0.04^b^	<0.001
TC (mmol/L)	3.20±0.08^a^	2.92±0.04^b^	2.87±0.05^b^	2.90±0.08^b^	0.004
LDLC (mmol/L)	0.95±0.04^a^	0.68±0.03^c^	0.73±0.03^bc^	0.85±0.03^ab^	<0.001
HDLC (mmol/L)	1.77±0.06	1.85±0.04	1.86±0.06	1.79±0.07	0.642

Values are presented means±standard error of the means, n = 8.

TG, total triglyceride; TC, total cholesterol; LDLC, low density lipoprotein cholesterol; HDLC, high density lipoprotein cholesterol.

a–c Means within a row with different superscripts differ significantly (p<0.05).

**Table 6:** Effects of lycopene on hepatic enzymatic activity of broiler chickens

Items	Control	Lycopene (mg/kg)			p-value

		100	200	400	
FAS (U/g protein)	121.91±3.85^a^	112.44±5.30^ab^	105.07±5.09^ab^	99.88±5.35^b^	0.022
ACC (U/g protein)	25.43±0.99^a^	20.03±1.17^b^	18.46±0.87^b^	17.35±0.67^b^	<0.001
LPL (U/g protein)	19.48±1.20	20.32±1.00	20.55±0.69	18.79±1.05	0.591
HL (U/g protein)	68.17±2.89	69.40±2.43	70.06±3.35	72.54±3.22	0.770

Values are presented means±standard error of the means, n = 8.

FAS, fatty acid synthase; ACC, acetyl-CoA carboxylase; LPL, lipoprotein lipase; HL, hepatic lipase.

a,b Means within a row with different superscripts differ significantly (p<0.05).
